# Platelet Factor 4 Antibodies and Severe AKI

**DOI:** 10.34067/KID.0000000000000287

**Published:** 2023-11-01

**Authors:** Charlotte Thomas, Rafia Ali, Isabel Park, Helena Kim, Samuel Short, Sarah Kaunfer, Lavanya Durai, Osman A. Yilmam, Tushar Shenoy, Elisabeth M. Battinelli, Hanny Al-Samkari, David E. Leaf

**Affiliations:** 1 Harvard Medical School, Boston, Massachusetts; 2 Division of Renal Medicine, Brigham and Women's Hospital, Boston, Massachusetts; 3 Larner College of Medicine, University of Vermont, Burlington, Vermont; 4 Division of Hematology, Brigham and Women's Hospital, Boston, Massachusetts; 5 Division of Hematology, Massachusetts General Hospital, Boston, Massachusetts

**Keywords:** AKI, platelet factor 4, PF4, heparin-induced thrombocytopenia, thrombosis

## Abstract

**Key Points:**

- Patients testing positive for platelet factor 4 antibodies have a >50% higher odds of developing severe AKI compared with those who test negative.
- The relationship between platelet factor 4 antibodies and severe AKI was independent of demographics, comorbidities, laboratory values, and severity-of-illness characteristics.

**Background:**

Heparin-induced thrombocytopenia, which results from production of antibodies that bind to heparin-platelet factor 4 (PF4) complexes, is a hypercoagulable state associated with considerable morbidity and mortality due to thrombotic complications. We investigated whether PF4 antibodies are associated with an increased risk of AKI.

**Methods:**

We conducted a cohort study of hospitalized adults who underwent testing for PF4 antibodies at two large medical centers in Boston between 2015 and 2021. The primary exposure was PF4 test positivity. The primary outcome was severe AKI, defined by Kidney Disease: Improving Global Outcomes stage 3 as a ≥3-fold increase in serum creatinine or receipt of KRT within 7 days after the PF4 test. We used multivariable logistic regression to adjust for potential confounders.

**Results:**

A total of 4224 patients were included in our analysis, 469 (11.1%) of whom had a positive PF4 test. Severe AKI occurred in 50 of 469 patients (10.7%) with a positive PF4 test and in 235 of 3755 patients (6.3%) with a negative test (unadjusted odds ratio, 1.79 [95% confidence interval, 1.30 to 2.47]). In multivariable analyses adjusted for demographics, comorbidities, laboratory values, and severity-of-illness characteristics, PF4 test positivity remained associated with a higher risk of severe AKI (adjusted odds ratio, 1.56 [95% confidence interval, 1.10 to 2.20]).

**Conclusions:**

Among hospitalized adults, the presence of PF4 antibodies is independently associated with a 56% higher odds of developing severe AKI. Additional studies are needed to investigate potential mechanisms that may underlie these findings, such as pathogenic effects of PF4 antibodies on the microvasculature of the kidneys.

## Graphical abstract

**Figure:**
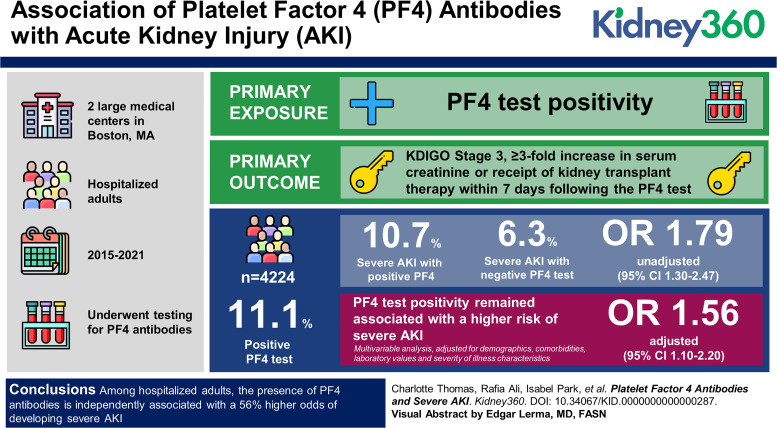

## Introduction

Heparin is one of the most commonly administered medications in hospitalized patients used both prophylactically and therapeutically to treat myriad conditions, including deep vein thrombosis (DVT), pulmonary embolism (PE), acute coronary syndrome, and others. Heparin-induced thrombocytopenia (HIT) is a well-documented and serious complication of heparin use, occurring in up to 3% of patients exposed to heparin.^1–3^ HIT occurs because of production of an IgG autoantibody directed against endogenous platelet factor 4 (PF4) complexed with heparin on the surface of platelets. Binding of these autoantibodies to heparin-PF4 complexes leads to platelet activation and release of additional PF4, which creates a positive feedback loop of further platelet activation.^4^

Patients who develop HIT are at high risk of venous and arterial thrombosis and associated sequelae, including skin necrosis, limb gangrene, and organ ischemia or infarction. These thrombotic manifestations have been primarily described in the macrovascular circulation.^5^ However, limited data suggest that HIT may also cause microvascular thrombosis.^6–8^

Because the kidney is a highly vascularized organ and renal microvascular thrombosis has been observed in multiple kidney diseases,^9–11^ we hypothesized that production of PF4 antibodies would be independently associated with higher risk of AKI in hospitalized patients.

## Methods

### Study Design

We conducted a cohort study of all adults who had a PF4 test obtained during an admission to Brigham and Women's Hospital or Massachusetts General Hospital (both in Boston, MA) between May 2015 and October 2021 (*N*=5476). All protocols were approved by the Mass General Brigham (MGB) Institutional Review Board.

### Study Cohort

**Figure 1:**
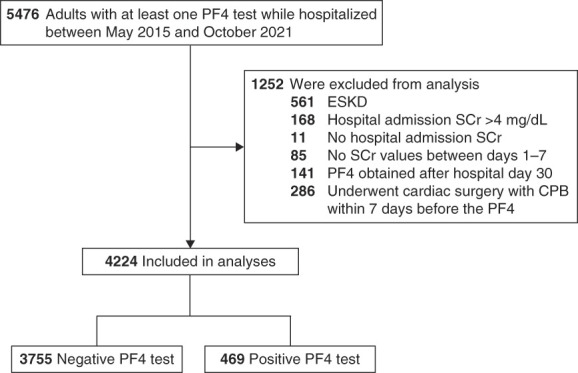
**Study cohort.** PF4, platelet factor 4; SCr, serum creatinine.

We derived our study cohort from the MGB Research Patient Data Registry, a central clinical data warehouse maintained by MGB. We excluded patients who had any of the following: (*1*) ESKD, (*2*) serum creatinine (SCr) >4 mg/dl at hospital admission, (*3*) absence of a SCr measurement at hospital admission, (*4*) absence of SCr measurement within 7 days after the PF4 test, (*5*) PF4 test was obtained >30 days after hospital admission, and (*6*) PF4 test was obtained within 7 days after cardiac surgery with cardiopulmonary bypass (because of the high risk of a false positive in this setting).^12^ After applying the abovementioned exclusion criteria, our final cohort consisted of 4224 patients (Figure 1).

### Primary Exposure

The primary exposure was the PF4 antibody immunoassay test (Immucor GTI Diagnostics, Inc. PF4 IgG ELISA), which was used by the laboratories at both hospitals during the period of interest. As recommended by the manufacturer, PF4 tests were considered positive if the optical density was ≥0.4 units and negative if <0.4. If a patient had more than one PF4 test obtained, the initial test was used, with the exception of patients who tested negative initially but then tested positive subsequently, in which case the first positive test was used.

### Primary and Secondary Outcomes

The primary outcome was severe AKI, defined as a ≥3-fold increase in SCr compared with the hospital admission value or receipt of KRT within 7 days after the PF4 test. This definition of AKI, consistent with modified Kidney Disease Improving Global Outcomes consensus criteria for stage 3 AKI,^13^ was selected as the primary outcome because it represents the most severe and clinically relevant form of AKI. We chose a 7-day window relative to PF4 testing to assess severe AKI because most thrombotic manifestations of HIT occur within this time window.^14^

Secondary outcomes included AKI of any stage (defined as a ≥50% increase in SCr compared with the hospital admission value or receipt of KRT within 7 days after the PF4 test); the composite of AKI or death within 7 days; thrombosis, including DVT, PE, ischemic stroke, and other arterial thrombosis; and a composite of any of the above thromboses, each assessed within 7 days of the PF4 test. Among PF4+ patients with severe AKI, we also assessed rates of renal recovery at hospital discharge, defined as survival to discharge, independent of KRT, and with SCr <1.5-fold the admission value.

### Data Collection

Using the Research Patient Data Registry, we collected data on demographics, comorbidities, procedures, laboratory values, severity of illness, and outcomes. Data were collected using International Classification of Diseases-9/10 diagnosis and procedure codes and Current Procedural Terminology codes. A complete list of diagnosis and procedure codes is provided in Supplemental Table 1. In addition, in a subcohort of 100 randomly selected patients (50 PF4-positive and 50 PF4-negative), we performed detailed chart review to assess for imbalances in characteristics that were not available for the entire cohort. Specifically, we obtained additional data on nephrotoxin exposure, laboratory values (C-reactive protein and lactate), hospital location at the time of PF4 testing (intensive care unit [ICU] versus non-ICU), type of heparin exposure (intravenous unfractionated heparin, subcutaneous unfractionated heparin, or subcutaneous low–molecular weight heparin), and heparin indication.

### Statistical Analysis

Data are shown as median (interquartile range [IQR]) and as count (%). We compared baseline characteristics between patients with positive versus negative PF4 tests using the Wilcoxon rank-sum and chi-squared tests for continuous and categorical variables, respectively.

Our primary analysis investigated whether patients with a positive PF4 test are at higher risk of severe AKI compared with patients with a negative PF4 test. We used univariate and multivariable logistic regression models to calculate odds ratios (ORs) and 95% confidence intervals (CIs) for risk of severe AKI in PF4-positive versus negative patients, using complete case analysis. The following covariates were prespecified on the basis of clinical knowledge,^15–17^ biologic plausibility, univariate associations, and parsimony: age; sex; race (White versus non-White); baseline eGFR (≥90; 60–89; 45–59; 30–44; <30 ml/min per 1.73 m^2^), defined using the closest SCr value within 7–365 days before hospital admission or, if unavailable, the trough value in the first 30 days of hospitalization,^18^ and with use of the 2021 CKD Epidemiology Collaboration equation^19^; comorbidities (hypertension, diabetes mellitus, congestive heart failure, chronic liver disease, and active malignancy); and severity of illness (white blood cell count, hemoglobin, and platelet count, invasive mechanical ventilation, sepsis, and shock). The abovementioned laboratory parameters were assessed on the day of the PF4 test, invasive mechanical ventilation was assessed within 2 days before the PF4 test, and sepsis and shock were assessed within 7 days before the PF4 test. Secondary outcomes were assessed using a similar modeling approach.

#### Sensitivity Analyses

We conducted three prespecified sensitivity analyses of the primary outcome. First, we assessed a composite outcome of severe AKI or death, to account for death as a competing risk of AKI. Second, we limited the analysis to patients who did not receive KRT between hospital admission and the day of the PF4 test. Third, we limited the analysis to patients with a PF4 test performed within the first 10 days of hospital admission.

#### Subgroup Analyses

In exploratory analyses, we used similar methods as the primary analysis to assess the association between PF4 test positivity and severe AKI across the following prespecified subgroups: age (<65 versus ≥65 years); sex; baseline eGFR (<60 versus ≥60 ml/min per 1.73 m^2^); platelet count on the day of the PF4 test (>100, 50–100, and <50 K/*µ*l); invasive mechanical ventilation within 2 days before the PF4 test; sepsis or shock within 7 days before the PF4 test; and thrombosis within 7 days of the PF4 test. We compared differences among subgroups by adding product (interaction) terms between the subgroup variable and the PF4 test group into the multivariable model.

All comparisons are two tailed, with *P* < 0.05 considered significant. Analyses were performed using SQL in Microsoft Access (Microsoft Access for Microsoft 365 MSO) and SAS version 9.4 (Cary, NC).

## Results

### Baseline Characteristics

The initial study population included 5476 patients. After excluding 1252 patients (as described in the Methods), the final study cohort consisted of 4224 patients, of whom 469 (11.1%) had a positive PF4 test and 3755 (88.9%) had a negative test (Figure 1). PF4 tests were sent at a median of 5 days (IQR, 2–9) after hospital admission.

Baseline characteristics stratified by PF4 test positivity are summarized in Table 1. The median age was 65 years (IQR, 54–73), 2369 (56.1%) were men, and 3352 (79.4%) were White. Patients with positive and negative PF4 tests had similar distributions of demographic characteristics and most comorbidities, although patients with a positive PF4 test were less likely to have active malignancy and more likely to have congestive heart failure and a history of DVT, PE, and arterial thrombosis compared with patients with a negative PF4 test. In addition, PF4-positive patients had higher white blood cell counts, had lower hemoglobin, and were more likely to be invasively mechanically ventilated compared with PF4-negative patients. PF4-positive patients had modestly lower platelet counts compared with PF4-negative patients (median 68.5 [IQR, 50–98] versus 75 [IQR, 54–101] K/*µ*l), although the trajectories of the platelet counts were similar between the groups (Supplemental Figure 1).

**Table 1:** Patient characteristics at baseline

Characteristic	All Patients (*n*=4224)	PF4-Positive Patients (*n*=469)	PF4-Negative Patients (*n*=3755)	*P* Value
**Demographics**				
Age, yr, median (IQR)	65 (54–73)	64 (55–72)	65 (54–74)	0.38
Male sex, no. (%)	2369 (56.1)	263 (56.1)	2106 (56.1)	0.99
Race, *no.* (%)				0.85
*Asian*	136 (3.2)	17 (3.6)	119 (3.2)	
*Black*	285 (6.7)	36 (7.7)	249 (6.6)	
*White*	3352 (79.4)	365 (77.8)	2987 (79.5)	
*Other/more than one race*	13 (0.3)	2 (0.4)	11 (0.3)	
*Unknown/not reported*	438 (10.4)	49 (10.4)	389 (10.4)	
**Comorbidities**, ***no.* (%)**				
Any	3726 (88.2)	421 (89.8)	3305 (88.0)	0.29
*Hypertension*	2492 (59.0)	273 (58.2)	2219 (59.1)	0.73
*Diabetes mellitus*	1173 (27.8)	137 (29.2)	1036 (27.6)	0.48
*COPD or emphysema*	671 (15.9)	66 (14.1)	605 (16.1)	0.28
*Baseline eGFR, ml/min per 1.73 m*^*2*^*^a^*				0.04
≥90	2166 (51.3)	264 (56.3)	1902 (50.7)	
60–89	1160 (27.5)	110 (23.5)	1050 (28.0)	
45–59	436 (10.3)	39 (8.3)	397 (10.6)	
30–44	332 (7.9)	44 (9.4)	288 (7.7)	
<30	130 (3.1)	12 (2.6)	118 (3.1)	
*Hyperlipidemia*	1929 (45.7)	202 (43.1)	1727 (46.0)	0.24
*Peripheral arterial disease*	740 (17.5)	83 (17.7)	657 (17.5)	0.90
*Coronary artery disease*	1323 (31.3)	138 (29.4)	1185 (31.6)	0.37
*CHF*	1647 (39.0)	204 (43.5)	1443 (38.4)	0.04
*STEMI/NSTEMI*	625 (14.8)	78 (16.6)	547 (14.6)	0.24
*Atrial fibrillation or atrial flutter*	1521 (36.0)	180 (38.4)	1341 (35.7)	0.26
*Chronic liver disease*	1328 (31.4)	136 (29.0)	1192 (31.7)	0.25
*Active malignancy*	1453 (34.4)	116 (24.7)	1337 (35.6)	<0.001
*Prior thrombosis^b^*				
Ischemic stroke	467 (11.1)	61 (13.0)	406 (10.8)	0.16
DVT	558 (13.2)	114 (24.3)	444 (11.8)	<0.001
PE	445 (10.5)	89 (19.0)	356 (9.5)	<0.001
Arterial thrombosis	146 (3.5)	25 (5.3)	121 (3.2)	0.02
**Laboratory values, median (IQR)^c^**				
WBC count, per mm	9.1 (6.0–13.5)	11.0 (7.9–15.8)	8.8 (5.8–13.3)	<0.001
Hemoglobin, g/dl	9.1 (8.1–10.6)	9.0 (8.0–10.3)	9.1 (8.1–10.6)	0.04
Platelet count, K/*µ*l	74 (53–101)	68.5 (50–98)	75 (54–101)	0.03
Albumin, g/dl	2.9 (2.4–3.3)	2.8 (2.4–3.2)	2.9 (2.4–3.3)	0.25
Sodium, mmol/L	139 (136–142)	138 (135–142)	139 (136–142)	0.17
Potassium, mmol/L	4.1 (3.8–4.4)	4.1 (3.8–4.4)	4.1 (3.8–4.4)	0.77
Calcium, mg/dl	8.4 (8.0–8.8)	8.4 (8.0–8.9)	8.4 (8.0–8.8)	0.06
Blood urea nitrogen, mg/dl	23 (14–37)	24 (14–43)	23 (14–36)	0.10
Creatinine, mg/dl	1.01 (0.7–1.6)	1.03 (0.7–1.7)	1.00 (0.7–1.5)	0.14
**Severity of illness, *no.* (%)**				
Invasive mechanical ventilation^d^	286 (6.8)	48 (10.2)	238 (6.3)	0.002
Sepsis^e^	863 (20.4)	83 (17.7)	780 (20.8)	0.13
Shock^e^	1398 (33.1)	162 (34.5)	1236 (32.9)	0.50

The corresponding International Classification of Disease 9/10 codes of the comorbidities are provided in Supplemental Table 1. CHF, congestive heart failure; COPD, chronic obstructive pulmonary disease; DVT, deep vein thrombosis; IQR, interquartile range; PE, pulmonary embolism; PF4, platelet factor 4; WBC, white blood cell.

Data regarding white blood cell count were missing for 27 patients (0.6%).

Data regarding hemoglobin were missing for 28 patients (0.7%).

Data regarding platelet count were missing for 30 patients (0.7%).

Data regarding albumin were missing for 937 patients (22.2%).

Data regarding sodium were missing for 37 patients (0.9%).

Data regarding potassium were missing for 42 patients (1.0%).

Data regarding calcium were missing for 38 patients (0.9%).

Data regarding blood urea nitrogen were missing for 36 patients (0.9%).

Data regarding creatinine were missing for 32 patients (0.8%).

All other data are complete.

a Baseline eGFR was determined using the closest serum creatinine value within 7–365 days beforeadmission or, if unavailable, the trough value during the first 30 days of hospitalization. eGFR was calculated using the 2021 CKD Epidemiology Collaboration serum creatinine equation.^19^

b Refers to deep vein thrombosis, pulmonary embolism, ischemic stroke, and other arterial thrombosis that occurred more than 7 days before the platelet factor 4 test.

c Closest value within 2 days before the platelet factor 4 test.

d Assessed within 2 days before the platelet factor 4 test.

e Assessed within 7 days before the platelet factor 4 test.

In a subcohort of 100 randomly selected patients, additional characteristics, including nephrotoxin exposure, ICU location at the time of PF4 testing, medical versus surgical primary presenting problem, type of heparin exposure, and heparin indication, were similarly distributed between PF4-positive versus negative patients (Supplemental Table 2).

### Primary Outcome: Severe AKI

**Figure 2:**
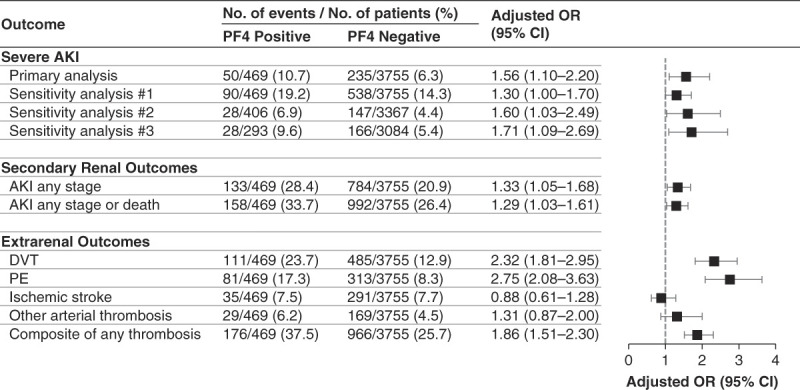
**Association between PF4 test positivity and severe AKI.** Multivariable models are adjusted for age, sex, race, baseline eGFR, hypertension, diabetes mellitus, CHF, chronic liver disease, active malignancy, laboratory values (WBC count, hemoglobin, and platelet count, each assessed on the day of the PF4 test), invasive mechanical ventilation, sepsis, and shock. Invasive mechanical ventilation was assessed within 2 days before the PF4 test, and sepsis and shock were assessed within 7 days before the PF4 test. Sensitivity analysis 1 assessed a composite outcome of severe AKI or death within 7 days after the PF4 test. Sensitivity analysis 2 was limited to patients who did not receive KRT between hospital admission and the day of the PF4 test. Sensitivity analysis 3 was limited to patients with a PF4 test performed within the first 10 days of hospital admission. DVT, PE, ischemic stroke, and other arterial thrombosis were each assessed within 7 days of the PF4 test. CHF, congestive heart failure; CI, confidence interval; DVT, deep vein thrombosis; PE, pulmonary embolism; WBC, white blood cell.

Severe AKI occurred in 50 of 469 patients (10.7%) who tested positive for PF4 and in 235 of 3755 patients (6.3%) who tested negative (unadjusted OR, 1.79 [95% CI, 1.30 to 2.47]). In a multivariable model adjusted for demographics, comorbidities, laboratory values, and severity of illness, PF4 test positivity remained significantly associated with a higher risk of severe AKI (adjusted OR, 1.56 [95% CI, 1.10 to 2.20]; Figure 2). The median time from hospital admission to development of severe AKI was 8 days (IQR, 3–11) and 6 days (IQR, 3–11) among PF4+ and PF4− patients, respectively. The median time from PF4 testing to development of severe AKI was 1 day (IQR, 0–1) in both groups.

The results were similar in all three sensitivity analyses. Specifically, PF4-positive patients had a higher adjusted OR of severe AKI in analyses assessing a composite of severe AKI or death (adjusted OR, 1.30 [95% CI, 1.00 to 1.70]), in analyses limited to patients who did not initiate KRT before the PF4 test (adjusted OR, 1.60 [95% CI, 1.03 to 2.49]), and in analyses limited to patients with a PF4 test performed in the first 10 days of hospitalization (adjusted OR, 1.71 [95% CI, 1.09 to 2.69]) (Figure 2). We did not observe an association between higher optical density and greater risk of severe AKI (Supplemental Table 3).

### Subgroup Analyses

The association between PF4 test positivity and severe AKI was similar across each of the following subgroups: age (≥65 versus <65 years; *P* = 0.11 for interaction), baseline eGFR (<60 versus ≥60 ml/min per 1.73 m^2^; *P* = 0.09 for interaction), platelet count (>100, 50–100, <50 K/*µ*l; *P* = 0.80 for interaction), invasive mechanical ventilation (*P* = 0.36 for interaction), sepsis (*P* = 0.54 for interaction), and shock (*P* = 0.72 for interaction). There was a trend toward a larger association between PF4 test positivity and severe AKI in men versus women (*P* = 0.07 for interaction) (Supplemental Figure 2).

### Secondary Outcomes

#### AKI of Any Stage

AKI of any stage occurred in 133 of 469 patients (28.4%) who tested positive for PF4 and in 784 of 3755 patients (20.9%) who tested negative (OR, 1.50 [95% CI, 1.21 to 1.86]). In multivariable models, PF4 test positivity remained significantly associated with a higher OR of AKI (adjusted OR, 1.33 [95% CI, 1.05 to 1.68]). The results were similar in analyses assessing a composite of AKI of any stage or death (Figure 2).

#### Thrombosis and Hospital Mortality

DVT, PE, arterial thrombosis, and hospital death each occurred more frequently in PF4-positive versus PF4-negative patients (Figure 2). In multivariable models, PF4 test positivity was associated with a higher risk of DVT (adjusted OR, 2.32 [95% CI, 1.81 to 2.95]), PE (adjusted OR, 2.75 [95% CI, 2.08 to 3.63]), and a composite of any thrombosis (adjusted OR, 1.86 [95% CI, 1.51 to 2.30]). PF4 test positivity was not associated with risk of ischemic stroke or other arterial thrombosis (Figure 2).

#### Renal Recovery

A total of ten of 50 PF4+ patients with severe AKI (20%) had renal recovery at hospital discharge. Patients with versus without renal recovery had similar rates and timing of heparin discontinuation following the PF4+ test result as well as similar timing of initiation of an alternative anticoagulant (Supplemental Table 4).

## Discussion

In this study of 4224 adult inpatients tested for PF4, we found that those with a positive test had a 56% higher odds of developing severe AKI compared with those with a negative test. This independent relationship between PF4 test positivity and severe AKI was demonstrated in multivariable models adjusted for demographics, comorbidities, laboratory values, and severity-of-illness characteristics. The results were similar in multiple sensitivity analyses, including analyses that assessed a composite outcome of severe AKI or death.

Other than isolated case reports demonstrating a link between HIT and renal vein thrombosis,^20,21^ to the best of our knowledge, this is the first study to demonstrate an association between PF4 antibodies and AKI. Our findings are consistent with prior studies demonstrating that microvascular thrombosis is an important component of AKI pathogenesis across a variety of clinical settings.^22–25^ Our findings are also consistent with prior studies that reported thrombosis of both large^5^ and small vessels^6–8^ in patients with HIT.

The independent association that we found between PF4 antibodies and a higher risk of severe AKI has important clinical implications. First, it underscores the importance of timely recognition and treatment of HIT. Second, it suggests that patients with a positive PF4 test should be closely monitored for the development of AKI, perhaps with implementation of preventative strategies, such as the Kidney Disease Improving Global Outcomes bundle,^26^ or with more personalized strategies for kidney protection.^27^ Third, our findings suggest that hypercoagulability and the formation of platelet-rich microthrombi in the kidneys could be an important therapeutic target for AKI prevention in certain clinical settings associated with a hypercoagulable state. Therapeutic-dose anticoagulation as compared with usual-care thromboprophylaxis was recently demonstrated in a multicenter randomized clinical trial to increase the probability of survival to hospital discharge without organ support among noncritically ill hospitalized patients with coronavirus disease 2019.^28^ A secondary analysis of these data found that therapeutic-dose anticoagulation may also reduce the incidence of AKI,^29^ although confirmation of these findings is needed.

Our results were consistent across multiple subgroups. Interestingly, we observed a greater association between PF4 test positivity and severe AKI in men compared with women. Important differences in platelet count, function, and activation have been observed in men and women for nearly half a century.^30,31^ A recent study found that thrombin-mediated platelet activation is augmented in men compared with women at the time of myocardial infarction.^32^ By contrast, women may be at higher risk of HIT-associated thrombotic events and death compared with men,^33,34^ although not all studies have demonstrated this relationship.^35^ Whether men are more predisposed than women to PF4-mediated microthrombotic sequalae, such as AKI, is an intriguing possibility that requires further study. Alternatively, our observations may be due to a type 1 error due to the multiple subgroups that were assessed.

We acknowledge several limitations. First, we used diagnostic and procedure codes to define comorbidities and procedures; however, the primary outcome of severe AKI was determined with the use of daily SCr data. Second, data on urine output were unavailable, and thus, AKI was determined according to changes in SCr and receipt of KRT only. Third, although there was a clinical suspicion for HIT in all patients analyzed because PF4 testing was a requirement for inclusion in the study, clinical data regarding HIT, such as the 4Ts score,^36^ were unavailable. Fourth, data for this study came from patients admitted to two hospitals in Boston and may not be fully generalizable to other populations. In particular, approximately 80% of the patients included were White, and Black patients may have been underrepresented. Fifth, although our multivariable models were adjusted for a comprehensive set of covariates, we cannot exclude the possibility of residual confounding, as with any observational study. However, we manually reviewed the charts of 100 randomly selected patients to ascertain data on clinical variables that were not available for the entire cohort and found their distribution to be similar between PF4-positive versus negative patients (Supplemental Table 2). Sixth, severe AKI was defined as a ≥3-fold increase in SCr compared with the hospital admission value or initiation of KRT within 7 days after the PF4 test. Accordingly, AKI onset could have occurred before PF4 testing because our exposure of interest was the development of PF4 antibodies, which precedes clinical suspicion (and thus diagnostic testing) for it. However, in sensitivity analysis 2, we excluded patients who initiated KRT before the PF4 test, and the results were similar to the primary analyses (Figure 2). Finally, although we excluded patients whose PF4 test was obtained within 7 days after cardiopulmonary bypass surgery due to the high risk of false positives in this setting, we cannot exclude the possibility of false-positive results from other causes; however, the presence of false-positive results would have only biased our results toward the null.

In conclusion, we found that PF4 test positivity is independently associated with severe AKI among hospitalized adult patients. These findings should prompt clinicians to closely monitor kidney function in patients with PF4 antibodies and minimize exposure to nephrotoxins and other renal insults. Further mechanistic studies are needed to investigate whether PF4 antibodies are truly pathogenic to the kidneys, and whether targeting a hypercoagulable state could be a therapeutic target for AKI prevention in certain clinical settings.

## Data Availability

Anonymized data created for the study are or will be available in a persistent repository upon publication. Observational Data; Raw Data/Source Data. Other. Mendeley's Digital Common Data. The data have not yet been uploaded to Mendeley. They will be uploaded following publication of the paper.

## Supplemental Material

Supplemental Table 1. Diagnosis and procedures codes.

Supplemental Table 2. Additional characteristics assessed in a random sample of 50 PF4+ and 50 PF4− patients.

Supplemental Table 3. PF4 optical density and incidence of severe AKI.

Supplemental Table 4. Characteristics of PF4+ patients with severe AKI with versus without renal recovery.

Supplemental Figure 1. Platelet count trajectory in PF4-positive and negative patients.

Supplemental Figure 2. Association between PF4 test positivity and severe AKI across subgroups.

## References

[1] Martel N Lee J Wells PS. Risk for heparin-induced thrombocytopenia with unfractionated and low-molecular-weight heparin thromboprophylaxis: a meta-analysis. Blood. 2005;106(8):2710–2715. doi:10.1182/blood-2005-04-1546 15985543

[2] Pohl C Kredteck A Bastians B Hanfland P Klockgether T Harbrecht U. Heparin-induced thrombocytopenia in neurologic patients treated with low-molecular-weight heparin. Neurology. 2005;64(7):1285–1287. doi:10.1212/01.WNL.0000156947.45112.16 15824368

[3] Warkentin TE Levine MN Hirsh J, . Heparin-induced thrombocytopenia in patients treated with low-molecular-weight heparin or unfractionated heparin. N Engl J Med. 1995;332(20):1330–1335. doi:10.1056/NEJM199505183322003 7715641

[4] Rollin J Pouplard C Gruel Y. Risk factors for heparin-induced thrombocytopenia: focus on Fcγ receptors. Thromb Haemost. 2016;116(5):799–805. doi:10.1160/TH16-02-0109 27358188

[5] Arepally GM Padmanabhan A. Heparin-induced thrombocytopenia: a focus on thrombosis. Arterioscler Thromb Vasc Biol. 2021;41(1):141–152. doi:10.1161/ATVBAHA.120.315445 33267665 PMC7769912

[6] Warkentin TE Roberts RS Hirsh J Kelton JG. Heparin-induced skin lesions and other unusual sequelae of the heparin-induced thrombocytopenia syndrome: a nested cohort study. Chest. 2005;127(5):1857–1861. doi:10.1378/chest.127.5.1857 15888871

[7] Segna E Bolzoni AR Baserga C Baj A. Free flap loss caused by heparin-induced thrombocytopenia and thrombosis (HITT): a case report and literature review. Acta Otorhinolaryngol Ital. 2016;36(6):527–533. doi:10.14639/0392-100X-1188 28177337 PMC5317135

[8] Blank M Shoenfeld Y Tavor S, . Anti-platelet factor 4/heparin antibodies from patients with heparin-induced thrombocytopenia provoke direct activation of microvascular endothelial cells. Int Immunol. 2002;14(2):121–129. doi:10.1093/intimm/14.2.121 11809731

[9] George JN Nester CM. Syndromes of thrombotic microangiopathy. N Engl J Med. 2014;371(7):654–666. doi:10.1056/NEJMra1312353 25119611

[10] Nath KA Hebbel RP. Sickle cell disease: renal manifestations and mechanisms. Nat Rev Nephrol. 2015;11(3):161–171. doi:10.1038/nrneph.2015.8 25668001 PMC4701210

[11] Rapkiewicz AV Mai X Carsons SE, . Megakaryocytes and platelet-fibrin thrombi characterize multi-organ thrombosis at autopsy in COVID-19: a case series. EClinicalMedicine. 2020;24:100434. doi:10.1016/j.eclinm.2020.100434 32766543 PMC7316051

[12] Welsby IJ Krakow EF Heit JA, . The association of anti-platelet factor 4/heparin antibodies with early and delayed thromboembolism after cardiac surgery. J Thromb Haemost. 2017;15(1):57–65. doi:10.1111/jth.13533 27714919 PMC5280211

[13] KDIGO clinical practice guideline for acute kidney injury. Kidney Int Suppl. 2012; 2(1):1–138.

[14] Warkentin TE Sheppard JA Moore JC Cook RJ Kelton JG. Studies of the immune response in heparin-induced thrombocytopenia. Blood. 2009;113(20):4963–4969. doi:10.1182/blood-2008-10-186064 19144981

[15] Martin-Cleary C Molinero-Casares LM Ortiz A Arce-Obieta JM. Development and internal validation of a prediction model for hospital-acquired acute kidney injury. Clin Kidney J. 2021;14(1):309–316. doi:10.1093/ckj/sfz139 33564433 PMC7857831

[16] Mehta RL Burdmann EA Cerdá J, . Recognition and management of acute kidney injury in the International Society of Nephrology 0by25 Global Snapshot: a multinational cross-sectional study. Lancet. 2016;387(10032):2017–2025. doi:10.1016/S0140-6736(16)30240-9 27086173

[17] Uchino S Kellum JA Bellomo R, . Acute renal failure in critically ill patients: a multinational, multicenter study. JAMA. 2005;294(7):813–818. doi:10.1001/jama.294.7.813 16106006

[18] Siew ED Ikizler TA Matheny ME, . Estimating baseline kidney function in hospitalized patients with impaired kidney function. Clin J Am Soc Nephrol. 2012;7(5):712–719. doi:10.2215/CJN.10821011 22422536 PMC3338282

[19] Inker LA Eneanya ND Coresh J, . New creatinine- and cystatin C-based equations to estimate GFR without race. N Engl J Med. 2021;385(19):1737–1749. doi:10.1056/NEJMoa2102953 34554658 PMC8822996

[20] Klompas AM Albright RC Maltais S Demirci O. Acute renal failure due to bilateral renal vein thromboses: a rare complication of heparin-induced thrombocytopenia. Ann Card Anaesth. 2019;22(2):204–206. doi:10.4103/aca.ACA_114_18 30971604 PMC6489386

[21] Somers DL Sotolongo C Bertolatus JA. White clot syndrome associated with renal failure. J Am Soc Nephrol. 1993;4(2):137–141. doi:10.1681/ASN.V42137 8400075

[22] Krishnan S Suarez-Martinez AD Bagher P, . Microvascular dysfunction and kidney disease: challenges and opportunities? Microcirculation. 2021;28(3):e12661. doi:10.1111/micc.12661 33025626 PMC9990864

[23] Sutton TA Fisher CJ Molitoris BA. Microvascular endothelial injury and dysfunction during ischemic acute renal failure. Kidney Int. 2002;62(5):1539–1549. doi:10.1046/j.1523-1755.2002.00631.x 12371954

[24] Matthys E Patton MK Osgood RW Venkatachalam MA Stein JH. Alterations in vascular function and morphology in acute ischemic renal failure. Kidney Int. 1983;23(5):717–724. doi:doi:10.1038/ki.1983.84 6876567

[25] Scrascia G Rotunno C Simone S, . Acute kidney injury in high-risk cardiac surgery patients: roles of inflammation and coagulation. J Cardiovasc Med. 2017;18(5):359–365. doi:10.2459/JCM.0000000000000343 26657082

[26] Meersch M Schmidt C Hoffmeier A, . Prevention of cardiac surgery-associated AKI by implementing the KDIGO guidelines in high risk patients identified by biomarkers: the PrevAKI randomized controlled trial. Intensive Care Med. 2017;43(11):1551–1561. doi:10.1007/s00134-016-4670-3 28110412 PMC5633630

[27] James MT Har BJ Tyrrell BD, . Effect of clinical decision support with audit and feedback on prevention of acute kidney injury in patients undergoing coronary angiography: a randomized clinical trial. JAMA. 2022;328(9):839–849. doi:10.1001/jama.2022.13382 36066520 PMC9449791

[28] Lawler PR Goligher EC Berger JS, . The ATTACC ACTIV-4a and REMAP-CAP Investigators. Therapeutic anticoagulation with heparin in noncritically ill patients with Covid-19. N Engl J Med. 2021;385(9):790–802. doi:10.1056/nejmoa2105911 34351721 PMC8362594

[29] Smilowitz NR Hade EM Kornblith L, . Effect of therapeutic-dose heparin on acute kidney injury in noncritically ill patients hospitalized for COVID-19. Res Pract Thromb Haemost. 2023;7(6):e102167. doi:10.1016/j.rpth.2023.102167 10.1016/j.rpth.2023.102167 PMC10506136 37727846

[30] Johnson M Ramey E Ramwell PW. Sex and age differences in human platelet aggregation. Nature. 1975;253(5490):355–357. doi:10.1038/253355a0 1110780

[31] Stevens RF Alexander MK. A sex difference in the platelet count. Br J Haematol. 1977;37(2):295–300. doi:10.1111/j.1365-2141.1977.tb06847.x 603762

[32] Soo Kim B Auerbach DS Sadhra H, . Sex-specific platelet activation through protease-activated receptors reverses in myocardial infarction. Arterioscler Thromb Vasc Biol. 2021;41(1):390–400. doi:10.1161/ATVBAHA.120.315033 33176447 PMC7770120

[33] Schenk S El-Banayosy A Prohaska W, . Heparin-induced thrombocytopenia in patients receiving mechanical circulatory support. J Thorac Cardiovasc Surg. 2006;131(6):1373–1381.e4. doi:10.1016/j.jtcvs.2006.01.048 16733172

[34] Thielmann M Bunschkowski M Tossios P, . Perioperative thrombocytopenia in cardiac surgical patients - incidence of heparin-induced thrombocytopenia, morbidities and mortality. Eur J Cardiothorac Surg. 2010;37(6):1391–1395. doi:10.1016/j.ejcts.2009.12.023 20138779

[35] Colarossi G Schnöring H Trivellas A, . Prognostic factors for patients with heparin-induced thrombocytopenia: a systematic review. Int J Clin Pharm. 2021;43(3):449–460. doi:10.1007/s11096-020-01166-2 33044680

[36] Lo GK Juhl D Warkentin TE Sigouin CS Eichler P Greinacher A. Evaluation of pretest clinical score (4 T's) for the diagnosis of heparin-induced thrombocytopenia in two clinical settings. J Thromb Haemost. 2006;4(4):759–765. doi:10.1111/j.1538-7836.2006.01787.x 16634744

